# Crocodile defensin (CpoBD13) antifungal activity via pH-dependent phospholipid targeting and membrane disruption

**DOI:** 10.1038/s41467-023-36280-y

**Published:** 2023-03-01

**Authors:** Scott A. Williams, Fung T. Lay, Guneet K. Bindra, Suresh Banjara, Ivan K. H. Poon, Thanh Kha Phan, Marc Kvansakul, Mark D. Hulett

**Affiliations:** Department of Biochemistry and Chemistry, La Trobe Institute for Molecular Science, La Trobe University, Melbourne, 3086 Australia

**Keywords:** X-ray crystallography, Innate immunity, Peptides

## Abstract

Crocodilians are an order of ancient reptiles that thrive in pathogen-rich environments. The ability to inhabit these harsh environments is indicative of a resilient innate immune system. Defensins, a family of cysteine-rich cationic host defence peptides, are a major component of the innate immune systems of all plant and animal species, however crocodilian defensins are poorly characterised. We now show that the saltwater crocodile defensin CpoBD13 harbors potent antifungal activity that is mediated by a pH-dependent membrane-targeting action. CpoBD13 binds the phospholipid phosphatidic acid (PA) to form a large helical oligomeric complex, with specific histidine residues mediating PA binding. The utilisation of histidine residues for PA engagement allows CpoBD13 to exhibit differential activity at a range of environmental pH values, where CpoBD13 is optimally active in an acidic environment.

## Abstract (web summary)

Defensins are a class of host defence peptides that contribute to the immune system of eukaryotes. Here, the authors report the structure of saltwater crocodile defensin CpoBD13 and the mechanism of pH-dependent antifungal activity.

## Introduction

Crocodilians are an order of ancient reptiles that have existed in their modern-day forms for approximately 83 million years^1^. During this time, these animals have evolved to thrive in microbe-laden environments. Yet despite regularly receiving wounds and even losing limbs in territorial disputes and interspecies conflicts, the development of infection is rare^2^. This unique ability to ward off pathogens before they can invade systemically is indicative of a formidable innate immune system^1^. A major constituent of an organism’s innate immune system are defensins; a family of cysteine-rich cationic host defence peptides (CHDPs) that harbor a broad range of activities, including direct microbicidal properties and inflammatory response signalling^3,4^. Defensins are grouped into two major superfamilies based on their intramolecular disulphide bonds; *cis*-defensins (expressed by plants, fungi and invertebrates) and *trans*-defensins (expressed by vertebrates and invertebrates)^4^. Within vertebrates, the *trans*-defensins are further subdivided into three sub-families termed the α-, β- and θ-defensins, with their distinguishing feature being the cysteine-pairing order of their disulphide bonds. While α- and θ-defensins are unique to mammals and old-world primates respectively, β-defensins are the most abundant and expressed by all vertebrates^4^.

β-defensins effectively inhibit and/or kill a wide range of pathogens including bacteria^5^, fungi^6^, viruses^7^ as well as tumourigenic cells^8^. This broad range of activities is primarily attributed to the destabilisation of the target cell plasma membrane, leading to death by lysis^3,9^. This mechanism of action was first characterised in plant defensins that were found to directly target and bind negatively charged phospholipids. Target phospholipids are typically members of the phosphatidylinositol phosphate (PIP) family and interact electrostatically with cationic residues of the defensin (typically arginine and lysine)^10,11^. Engagement of a target lipid by plant defensins triggers the formation of oligomeric defensin–lipid complexes which place torsional stress on the membrane until the point of lysis. Recently, this ability to target PIPs has been shown to be conserved in β-defensins, specifically human β-defensin 2 (HBD-2), which was able to kill the human pathogenic fungus *Candida albicans* through the binding of phosphatidylinositol 4,5-bisphosphate (PI(4,5)P_2_)^6^. While plant and human defensins have been studied in detail^6,8,10,11^, the defensins of other phyla, including reptiles, are poorly characterised.

We now report the functional and structural characterisation of *Crocodylus porosus* β-defensin 13 (CpoBD13). Our findings reveal that despite limited primary sequence identity, the three-dimensional structure of CpoBD13 bears a striking resemblance to HBD-2, suggesting strong evolutionary conservation of the β-defensin structure between humans and reptiles. Furthermore, we have also determined the structure of CpoBD13 bound to phosphatidic acid (PA), which identifies a continuous helical oligomer and reveals in atomic detail how *trans*-defensins are able to form large oligomeric membrane attack complexes. Importantly, we show that CpoBD13 permeabilises fungal cell membranes in a pH-dependent manner as a result of histidine-mediated binding of PA, revealing a mechanism to regulate defensin antimicrobial potency.

## Results

### CpoBD13 is a histidine-rich *trans*-defensin with pH-dependent antifungal activity

**Fig. 1:**
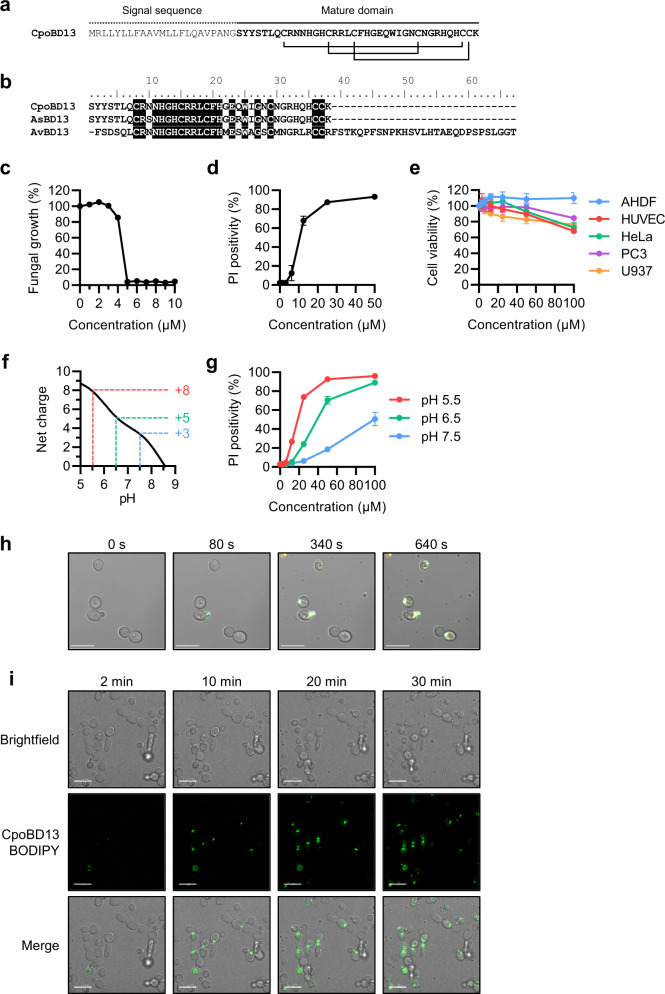
CpoBD13 is a histidine-rich β-defensin that permeabilises *C. albicans* in a pH-dependent manner. **a** Full-length peptide sequence of CpoBD13. Connecting lines represent disulphide bonds between cysteine residues. **b** Sequence alignment of CpoBD13 with its close homologues *A. sinensis* (Chinese alligator) β-defensin 13 (AsBD13, GenBank accession code AUG31287.1) and *Gallus gallus* (chicken) avian β-defensin 13 (AvBD13, NCBI RefSeq NP_001001780.1). **c** Fungal growth inhibition assay of *C. albicans* treated with CpoBD13 over a 24 h period. **d** Membrane permeabilisation of *C. albicans* after a 30 min treatment with CpoBD13 as determined by the uptake of propidium iodide. **e** MTT and MTS cell viability assay of human primary (AHDF and HUVEC) and tumourigenic (HeLa, PC3 and U937) cells treated with a range of CpoBD13 concentrations after 48 h. **f** Net charge of CpoBD13 at pH 5–9 highlights a sharp decrease in positive charge at pH greater than 6.0. The theoretical net charge of the peptide was calculated using a simplified method based on the pK_a_ of free amino acids. **g** Membrane permeabilisation of *C. albicans* treated with CpoBD13 in conditions buffered to pH 5.5, 6.5 and 7.5. Data in (c–e & g) represent mean ± SEM, *n* = 3. **h** Live microscopy of *C. albicans* treated with 20 µM CpoBD13 conjugated to the fluorophore BODIPY FL EDA (green) in the presence of PI (orange). Images are representative of three independent experiments. **i** Live microscopy of the accumulation of CpoBD13-BODIPY (20 µM, green) at the plasma membrane of *C. albicans* cells. Scale bars represent 10 µm. Images are representative of three independent experiments. (c–g) Source data are provided as a Source Data file.

To identify saltwater crocodile β-defensins, we searched *C. porosus* open reading frames acquired from the NCBI nucleotide database for potential defensin genes. CpoBD13, the homologue of avian β-defensin-13 and *Alligator sinensis* β-defensin-13, was selected for further characterisation based on its unusual amino acid sequence that featured a high proportion of histidine residues (Fig. 1a). While CpoBD13 has the defining characteristics of a typical β-defensin (e.g., six conserved cysteine residues, low molecular weight, positive net charge), the abundance of histidine residues is uncommon when compared to human defensins. These characteristics are however shared with close CpoBD13 homologues, AsBD13 and AvBD13 from the Chinese alligator and chicken, respectively (Fig. 1b).

Considering the known antifungal and anticancer activities of plant and human defensins, we examined the effect of recombinantly expressed CpoBD13 against the human pathogenic fungus *C. albicans* and an array of human primary and tumourigenic cell lines. In growth inhibition assays using *C. albicans*, CpoBD13 inhibited fungal growth in a concentration-dependent manner with an IC_50_ value of 4.1 µM (Fig. 1c). Furthermore, CpoBD13 was tolerant of salt and retained antifungal activity in NaCl concentrations of 100 mM (Supplementary Fig. 1a). To determine if the antifungal activity of CpoBD13 against *C. albicans* was a direct result of membrane perturbation, we performed flow cytometry-based membrane-permeabilisation assays monitoring the uptake of the membrane-impermeable dye PI (Fig. 1d). CpoBD13 permeabilised fungal cell membranes in a concentration-dependent manner. In contrast, CpoBD13 did not affect the viability of both primary and tumourigenic human cell lines in MTT and MTS assays at the assessed concentrations (Fig. 1e). The inability to kill tumourigenic cells was not a result of serum inactivation as CpoBD13 was unable to effectively permeabilise U937 cells in serum-free media (Supplementary Fig. 1b).

With five histidine residues present in its amino acid sequence (comprising ~13% of the mature sequence), the net charge of CpoBD13 is significantly altered by the pH of its surrounding environment (Fig. 1f). As the pK_a_ of histidine is 6.0, the charge of CpoBD13 can increase from +3 (at pH 7.5) to pH +8 (at pH 5.5), we surmised that changes in the net charge of CpoBD13 may impact its functionality. To assess if changes in the environmental pH modify the membrane-permeabilising activity of CpoBD13, we repeated the PI uptake assays in buffered media (Fig. 1g). At pH 5.5, the approximate pH of the standard assay in Fig. 1d, CpoBD13 was highly effective at permeabilising the fungal cells. As the pH increased to 6.5, however, CpoBD13 activity was significantly reduced, with the most dramatic loss of activity observed at pH 7.5. These results indicate that the ability for CpoBD13 to permeabilise fungal plasma membranes is pH-dependent and that optimal efficacy is achieved in an acidic environment.

To visualise fungal membrane-targeting and membrane permeabilisation, we treated *C. albicans* with fluorescently labelled CpoBD13 and observed the process by confocal microscopy (Fig. 1h). CpoBD13 accumulated at the fungal cell membrane before permeabilising the cell membrane, indicated by the appearance of PI fluorescence within the cell. Interestingly, for budding yeast and neighbouring cells in contact with one another, CpoBD13-BODIPY was often observed to accumulate at points of cell-cell contact (Fig. 1i, Supplementary Movie 1). Of the total cell to cell contact points in view, ~30% showed accumulation of CpoBD13-BODIPY (Supplementary Fig. 1c).

### CpoBD13 binds to and oligomerises with the phospholipid phosphatidic acid

**Fig. 2:**
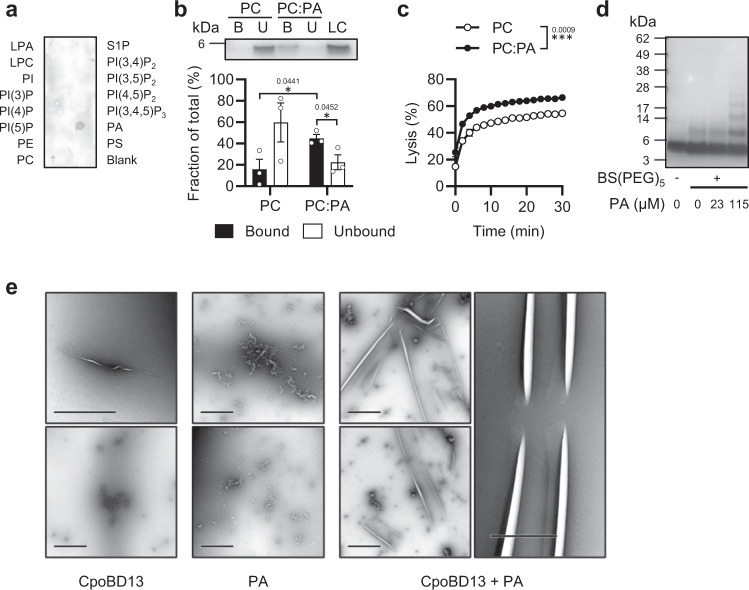
CpoBD13 binds and preferentially lyses liposomes containing the anionic phospholipid phosphatidic acid. **a** Immunodetection of CpoBD13-HA overlayed on a PIP Strip shows specific binding to PA. **b** Liposome pulldown comparing the binding of CpoBD13 to PC only and PC:PA (95:5 molar ratio) liposomes. Bound (B, black) and Unbound (U, white) fractions were subjected to SDS-PAGE analysis and colloidal Coomassie staining. The intensities of the bands were calculated as a fraction of a 1 µg loading control (LC) using ImageJ software. Data represent mean ± SEM, *n* = 3. *P < 0.05, two-tailed unpaired t test. **c** Liposome lysis of calcein-encapsulated PC only (white) and PC:PA (black) liposomes by 20 µM CpoBD13 over 30 min. Lysis was calculated as a fraction of a 100% lysis control using 0.1% Triton X-100. Data represent mean ± SEM, *n* = 3. ***P = 0.0009, two-way ANOVA. **d** Chemical crosslinking of CpoBD13 using BS(PEG)_5_ in the presence of various concentrations of PA followed by SDS-PAGE analysis and colloidal Coomassie staining. Image is representative of three independent experiments. **e** TEM imag**e**s of CpoBD13 alone, PA alone or CpoBD13:PA complexes (scale bars represent 1 µm). Images are representative of two independent experiments. (a–d) Source data are provided as a Source Data file.

The ability of human and plant defensins to target lipid bilayers has been attributed to direct phospholipid recognition and binding. To determine if CpoBD13 functions in the same manner, we assessed which phospholipids, if any, CpoBD13 interacts with. Haemagglutinin (HA)-tagged CpoBD13 in lipid overlay assays on PIP Strips (Fig. 2a) bound only to PA, with no clear binding detected for other lipids. We then examined CpoBD13 binding to PA in a physiologically relevant context by performing liposome-binding assays (Fig. 2b). Prepared phosphatidylcholine (PC) only and PC:PA liposomes were incubated with CpoBD13 before being separated into bound and unbound fractions. We found that CpoBD13 preferentially bound to PA-enriched liposomes, whereas only negligible binding to the PC only liposomes was observed. Furthermore, CpoBD13 lysed PC:PA liposomes as determined by measuring the release of calcein from calcein-encapsulated PC only and PC:PA liposomes (Fig. 2c). While PC only liposomes were also observed to lyse, there was a significant increase in the lysis of PC:PA liposomes.

The ability to bind and oligomerise with specific phospholipids has been well documented in other defensins, particularly those from plants, which belong to the *cis*-defensins and adopt large intricate protein:phospholipid oligomeric complexes. In contrast, whilst *trans*-defensins (such as those found in humans) have been shown to bind phospholipids, the atomic detail of putative large oligomeric defensin:phospholipid complexes remain to be determined^6,10,11^. Consequently, we subjected CpoBD13 to chemical crosslinking analysis in the presence and absence of PA (Fig. 2d). In the absence of the crosslinker BS(PEG)_5_ and PA, CpoBD13 is found primarily in a monomeric state. After the addition of BS(PEG)_5_, clear dimers are observed at 0 and 23 µM PA concentrations. At 115 µM PA, multiple higher order bands are clearly observable, indicative of multimeric complexes up to the size of pentamers. To visualise the morphology of these higher order complexes, samples of CpoBD13, PA only and CpoBD13:PA were examined by transmission electron microscopy (TEM) (Fig. 2e). CpoBD13:PA complexes formed large sheet-like structures that were vastly different to the fibrils that have been observed in plant defensins^10,11^. In contrast, for CpoBD13 alone these structures were absent, although smaller fragmented sheets were infrequently encountered. This indicates that these structures may form in the absence of PA, however, not to the same scale and extent. In contrast, no such structures were observed in PA-only samples. Additionally, CpoBD13 did not form any observable structures when paired with PC (Supplementary Fig. 2a). Further investigation into the structural stability of the defensin-lipid complexes revealed these structures are disrupted in reducing conditions (Supplementary Fig. 2b).

### CpoBD13–PA interactions are mediated by arginine and histidine residues

To understand the structural basis of CpoBD13 activity, we determined its atomic structure using X-ray crystallography. A single copy of CpoBD13 was found in the asymmetric unit, with clear and continuous density observed for the entire protein chain. CpoBD13 adopts a fold comprising the family-defining structural elements observed in mammalian β-defensins comprising a single α-helix and a three-stranded β-sheet, which are connected via three disulphide bonds. A Dali analysis of CpoBD13 revealed that human β-defensin-2 (HBD-2) is the closest structural homologue in the Protein Data Bank (PDB), with a rmsd of 0.8 Å (Z-score 6.2) over 40 Cα atoms, and a peptide sequence identity of 26% (only I26 and K38 are conserved in addition to the cysteine residues that form the structurally critical disulphide bonds).

**Fig. 3:**
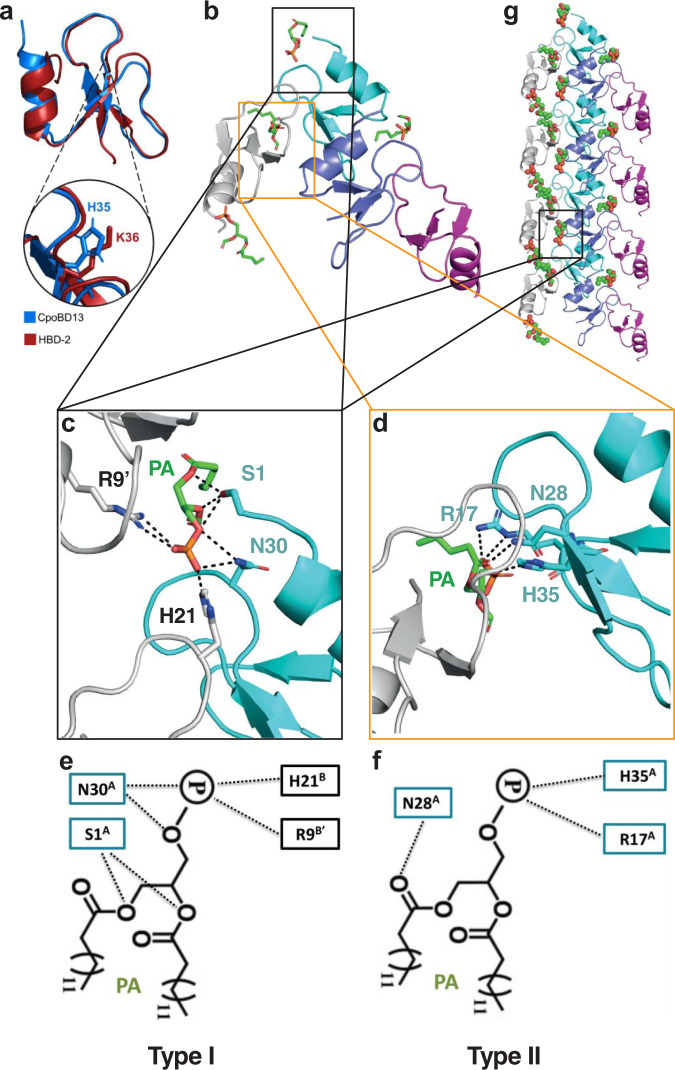
Crystal structure of the CpoBD13:PA complex. **a** Three-dimensional structure of CpoBD13 determined by X-ray crystallography superimposed with HBD-2 (PDB ID 1FD3). Zoomed in region shows the substitution of K36 (a crucial residue for membranolytic activity) in HBD-2 for H35 in CpoBD13. **b** Four CpoBD13 chains are found in the asymmetric unit (white, cyan, steel blue and magenta cartoon), bound to four PA molecules shown as green sticks. **c** Type I and **d** Type II PA binding sites. Key interacting residues are shown as sticks. Hydrogen bonds or ionic interactions are marked as black dashed lines. Schematic representation of the **e** Type I and **f** Type II PA binding sites. **g** CpoBD13:PA complex forms a single-stranded left-handed helix. The coil shown comprises the content of four asymmetric units. The location of a single type I PA binding site is boxed in black.

An overlay of the two defensin structures (Fig. 3a) revealed that residue H35 of CpoBD13 was located in the same position as the key lipid binding residue K36 in HBD-2. We surmised that if the activity of CpoBD13 is as conserved with HBD-2 as its tertiary structure, the co-localisation of these amino acids could indicate that histidine may play a role in CpoBD13–lipid interactions. However, considering the diversity of phospholipid binding sites found across defensins, CpoBD13 may not necessarily use a previously observed interaction mode with PA. Consequently, we determined the structure of a CpoBD13:PA complex, which was refined to a resolution of 1.45 Å. The asymmetric unit features four CpoBD13 protomers and four PA molecules (Fig. 3b). Examination of the mode of engagement of PA with the different CpoBD13 chains revealed the presence of two different PA binding sites. The type II site (Fig. 3d, f) involves contacts of R17 and H35 with the phosphate of the PA headgroup. An additional contact is formed with N28 and a carboxyl group of the acyl chain. Notably, all three contact residues are provided by a single CpoBD13 protomer.

In contrast, the type I site (Fig. 3c, e) features contributions from three different CpoBD13 chains. N30 from chain A and H21 from chain B contact the phosphate of the PA headgroup. Furthermore, the crystallographic symmetry mate of chain B (denoted as B’) also contributes via R9 binding to the PA headgroup. Three additional contacts are formed by S1 from chain A, which forms hydrogen bonds with two oxygen atoms in the acyl chains of PA and the linking oxygen with the phosphate headgroup. Whilst the asymmetric unit does not contain an obvious large oligomeric CpoBD13:PA complex (as observed using negative stain TEM (Fig. 2e), examination of the crystal lattice reveals that PA groups bound in type I sites make contacts across neighbouring asymmetric units, in the process creating a continuous left-handed single stranded helical chain of CpoBD13 protomers connected via PA molecules (Fig. 3g).

### The antifungal activity of CpoBD13 is dependent on PA-binding residues

**Fig. 4:**
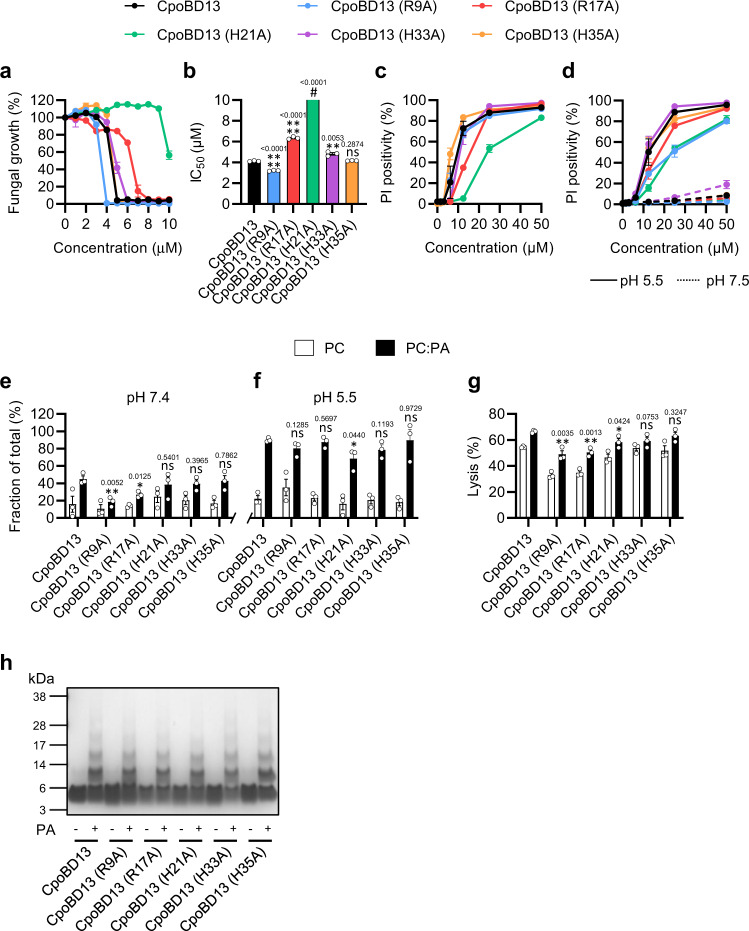
Structure–function analysis of the interaction between CpoBD13 and PA. Data represent ± SEM, *n* = 3. All statistical evaluations are comparisons to the corresponding CpoBD13 wild type sample. ns, not significant, *P < 0.05, **P < 0.01, ****P < 0.0001 two-tailed unpaired t test. Exact *P* values are stated above their respective graphs. **a** Fungal growth inhibition of *C. albicans* treated with wild type or mutant CpoBD13 over 24 h. **b** Calculated IC_50_ values of the fungal growth curves. # The IC_50_ of CpoBD13 (H21A) could not be determined within the assessed concentration range (0–10 µM). **c** Membrane permeabilisation of *C. albicans* treated with wild type or mutant CpoBD13 for 30 min as determined by the uptake of PI. **d** Membrane permeabilisation of *C. albicans* in pH buffered conditions (pH 5.5 = solid line, pH 7.5 = dashed line) treated with wild type or mutant CpoBD13 for 30 min. **e** Liposome pulldown quantification of the bound fractions of PC only (white) and 95:5 molar ratio PC:PA (black) liposomes incubated with 1 µg of CpoBD13 or its mutants performed at pH 7.4 and **f** again at pH 5.5. **g** Liposome lysis of calcein-encapsulated PC only and PC:PA liposomes by 20 µM wild type or mutant CpoBD13 over 30 min. Lysis was calculated as a fraction of a 100% lysis control using 0.1% Triton X-100. **h** Biochemical crosslinking of wild type or mutant CpoBD13 using BS(PEG)_5_ in the presence of PA followed by SDS-PAGE analysis and colloidal Coomassie staining. Image is representative of three independent experiments. (a–h) Source data are provided as a Source Data file.

To further understand the role of PA interactions for CpoBD13 activity, we performed structure-guided mutagenesis on key residues involved in the proposed type I (R9 and H21) and type II (R17 and H35) PA-binding sites and performed fungal growth inhibition assays using our panel of CpoBD13 mutants (Fig. 4a). We also mutated H33 to assess if losing a histidine residue outside of the binding sites had any effect on the function of CpoBD13.

No significant differences were observed for the activity of CpoBD13 (H35A) compared to the wild type and only minor changes in activities of CpoBD13 (R9A) and CpoBD13 (H33A) (Fig. 4b), whereas CpoBD13 (R17A) (IC_50_ of ~6 µM) and CpoBD13 (H21A) (IC_50_ could not be determined) both showed significant reductions in fungal growth inhibition activity. This pattern in activity was reflected in PI uptake assays with CpoBD13 (H21A) being the least effective at permeabilising *C. albicans* cell membranes followed by CpoBD13 (R17A) (Fig. 4c). In contrast, the remaining mutants displayed activity comparable to wild type CpoBD13. We then repeated these experiments at pH 5.5 and 7.5 to identify if any specific residues were important for regulating membranolytic activity at different pH values (Fig. 4d). Results showed that all CpoBD13 mutants were able to permeabilise fungal membranes at pH 5.5, however at pH 7.5 they were ineffective, indicating that the activities of all the peptides were still dependent on the environmental pH.

We then examined PA binding of our panel of mutants in liposome pulldown assays (Fig. 4e). Both arginine mutants showed a significant reduction in their PA binding capability whereas it was unchanged for the histidine mutants when compared to the wild type. At a pH of 5.5, the ability of CpoBD13 (R9A) and CpoBD13 (R17A) to bind PA was recovered with no significant difference observed compared to CpoBD13 **(**Fig. 4f**)**. CpoBD13 (H21A) was the least effective at binding PA at pH 5.5, whereas the remaining histidine mutants showed no significant differences to the wild type.

To establish if the differences in PA binding are reflected in the lytic activity of the mutants, we again treated PA-enriched liposomes and monitored calcein-release (Fig. 4g). The results of the liposome lysis followed the same trend observed in the pH 7.4 pulldown (Fig. 4e) with a significant decrease in lysis for CpoBD13 (R9A) and CpoBD13 (R17A), relative to wild type CpoBD13. In contrast, the histidine mutants showed no significant change, except for CpoBD13 (H21A), which showed a minor yet statistically significant decrease in activity. Lastly, we tested the ability of CpoBD13 mutants to oligomerise in the presence of PA (Fig. 4h) and found that there were no obvious differences in the formation of multimeric complexes across the panel of mutants.

### The pH sensitivity of CpoBD13 is controlled by both PA-interacting histidine residues working in combination

**Fig. 5:**
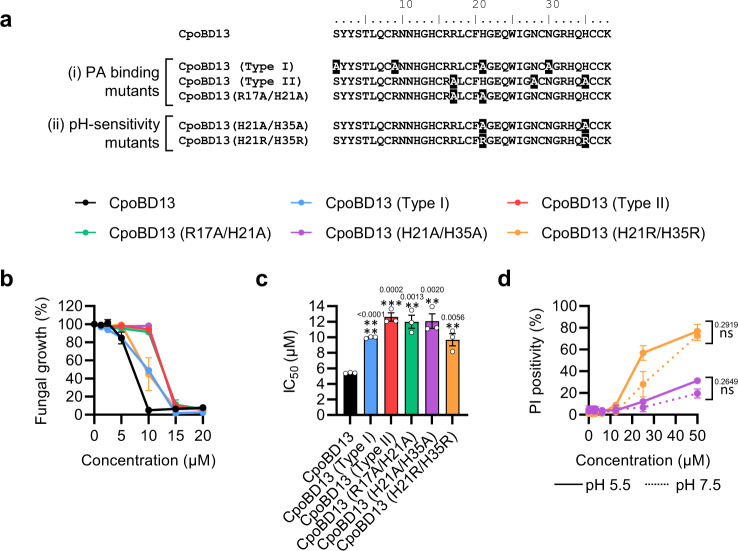
Effects of compound mutations in the PA-binding site of CpoBD13 on its antifungal activity. **a** Sequence alignment of CpoBD13 with the designed compound mutants. Residues that differ from wild type CpoBD13 are highlighted in black. **b** Fungal growth inhibition of *C. albicans* treated with a range of CpoBD13 and CpoBD13 compound mutant concentrations over 24 h. Data represent mean ± SEM, *n* = 3. **c** Calculated IC_50_ values of the fungal growth curves. Data represent mean ± SEM, *n* = 3. All statistical evaluations are comparisons to the corresponding CpoBD13 wild type sample. **P < 0.01, ***P < 0.001, ****P < 0.0001, two-tailed unpaired t test. Exact *P* values are stated above their respective graphs. **d** Membrane permeabilisation of *C. albicans* in pH-buffered conditions (pH 5.5 = solid line, pH 7.5 = dashed line) treated with CpoBD13 (H21A/H35A) (purple) and CpoBD13 (H21R/H35R) (orange) for 30 min. Data represent mean ± SEM, *n* = 3. ns, not significant, two-way ANOVA. (**b–d**) Source data are provided as a Source Data file.

As single amino acid substitutions of the PA-binding residues elicited mild changes in the antifungal activity of CpoBD13 (excluding H21A), we generated a range of compound mutations to further investigate the importance of PA interactions on the defensin’s ability to kill fungal cells. In total, five compound mutant defensins were recombinantly expressed to investigate two aspects of the defensin’s activities (Fig. 5a), the pH sensitivity and overall antifungal activity. Compound mutants were divided into two groups: the first group (i) included alanine substitutions of identified type I residues (CpoBD13 (Type I)) or type II residues (CpoBD13 (Type II)), and CpoBD13 (R17A/H21A) which contained alanine substitutions of the most important single residues (in terms of antifungal activity) from both binding pockets. These peptides were designed to determine if the defensin’s antifungal activity is a result of the identified PA binding residues working in combination. The second group (ii) included CpoBD13 (H21A/H35A) and CpoBD13 (H21R/H35R) which were designed to remove the charge or induce a persistent positive charge, respectively, in the position of the original histidine residues proposed to contact PA. These modifications would help in assessing if the pH-sensitivity of CpoBD13 was a result of the cumulative effect of both histidine residues.

To determine the consequence of each compound mutation on the antifungal activity of CpoBD13, we assessed their ability to inhibit the growth of *C. albicans* (Fig. 5b). There was a significant decrease in the growth inhibitory effect of all five compound mutations when comparing their respective IC_50_ values to wild type peptide (Fig. 5c). Both CpoBD13 (Type I) and CpoBD13 (H21R/H35R) showed an approximate 2-fold increase whereas, CpoBD13 (Type II), CpoBD13 (R17A/H21A) and CpoBD13 (H21A/H35A) presented a ~2.5-fold increase in IC_50._

As single amino acid substitutions had little effect on the pH sensitivity of CpoBD13, we assessed the ability of the double histidine substitution mutants CpoBD13 (H21A/H35A) and CpoBD13 (H21R/H35R) to permeabilise fungal cell membranes at pH 5.5 and 7.5 (Fig. 5d). At pH 5.5, CpoBD13 (H21A/H35A) was ineffective at killing *C. albicans*, with only ~30% of fungal cells permeabilised at the highest concentration tested. In contrast, CpoBD13 (H21R/H35R) had greater membranolytic activity and was able to permeabilise ~75% of the fungal cells at 50 µM. Furthermore, when the pH was adjusted to pH 7.5, neither mutant showed a significant difference in activity when compared to pH 5.5, indicating that the antifungal activities of the compound histidine mutants were not regulated by the surrounding pH.

## Discussion

The structure–function relationships of human and plant defensins have been well documented. These CHDPs target and destabilise the plasma membranes of a broad range of microbial organisms and, in certain cases, tumourigenic cells by interacting with negatively charged phospholipids^6,8,10^. While reptilian defensin genes have been identified from the genomes of species such as the Komodo dragon^12^, Chinese turtle^13^ and recently members of the crocodilian family^14,15^, the same depth of structural and mechanistic insight has not been obtained in comparison to their human and plant counterparts.

In this study, we characterised the antifungal activity of the saltwater crocodile β-defensin CpoBD13 and discovered that the ability to bind anionic phospholipids, specifically phosphatidic acid, has been evolutionarily conserved by reptilians. By solving the first three-dimensional crystal structure of a reptilian defensin in complex with PA, we identified that the binding of PA is mediated by key histidine residues that regulate the membranolytic activity of the peptide in a manner dependent on the environmental pH. To our knowledge, this phenomenon has not been observed for any characterised defensin.

CpoBD13 displayed antifungal activity against the model pathogenic yeast *C. albicans*, which is mediated via accumulation at the cell membrane prior to cell lysis (Fig. 1c, d, h and i). While some defensins also target tumourigenic cells^8,16,17^, CpoBD13 was not effective at reducing the viability of any of the mammalian cells assayed (Fig. 1e). Recently, a truncated form of CpoBD13 was shown to have minimal antibacterial activity against biofilms of the Gram-positive and Gram-negative bacteria, methicillin-resistant *Staphylococcus aureus* and *Salmonella enterica* serovar Typhimurium, respectively^18^. Collectively, these data suggest that CpoBD13 selectively targets fungal cells.

The antifungal activity of CpoBD13 is regulated by the environmental pH and has optimal activity at acidic pH. Its ability to permeabilise fungal membranes improves as the net charge of the peptide increases. While this is the first defensin determined to be pH-sensitive based on direct histidine-mediated lipid binding, the ability for pH to modulate membranolytic activity has been previously observed^19,20^. It has also been documented that histidine residues can regulate the activity of CHDPs. For example, by substituting integral lysine residues with histidine in the membranolytic peptide LL-1, the peptide was engineered to kill the human adenocarcinomic alveolar basal epithelial cells (A549) in a pH-dependent manner^21^. For CpoBD13, this in-built pH-sensitivity could be a mechanism that allows the peptide to be stored or transported in a low-activity state within the crocodile when maintained in a neutral pH to help prevent harm to the host. However, once CpoBD13 reached an acidic pH at the site of an infection (e.g. *Candida* blastophores favour acidic pH in humans^22^), the peptide could be activated and clear target pathogens.

The membranolytic activity of CpoBD13 is a result of binding PA within target cell plasma membranes, as demonstrated by its ability to bind and permeabilise PA-enriched liposomes (Fig. 2a–c). PA is an important signalling molecule involved in pH sensing and membrane fusion within budding yeast and accounts for 4% of the total plasma membrane^23^. Our crystal structure of CpoBD13 bound to PA (Fig. 3b) confirmed the presence of histidine residues within the proposed type I (Fig. 3c, e) and type II (Fig. 3d, f) PA-binding sites. We identified H21 as the most important residue for CpoBD13 antifungal activity (Fig. 4a–c) with H21 also involved in the pH-mediated binding of PA (Fig. 4f, g). The fungal growth inhibitory activity of CpoBD13 (H21A) resembled that of the compound mutant CpoBD13 (Type I), indicating that PA interactions through the type I site are largely reliant on H21 alone (Fig. 4a and Fig. 5b). While H21 is clearly important for CpoBD13 activity, CpoBD13 (H21A) retains some capacity to inhibit fungal growth and bind PA. Compound mutations in the type II PA-binding site and of R17/H21 decreased the antifungal activity of the defensin to a greater extent than their respective single amino acid substitutions alone, suggesting that PA binding residues are contributing collectively to the overall CpoBD13 activity (Fig. 5a–c). Furthermore, the pH-sensitivity of CpoBD13 is a result of H21 and H35 working in tandem. While alanine substitution of the individual residues did not alter the defensin’s ability to modify its activity based on the environmental pH (Fig. 4d), when both residues were substituted, the membranolytic activity of CpoBD13 (H21A/H35A) did not improve in an acidic environment (Fig. 5d). This was also true of CpoBD13 (H21R/H35R) however the inclusion of arginine residues in position 21 and 35 had the opposite effect to CpoBD13 (H21A/H35A), in that it was able to retain its membranolytic activity when the pH was increased from 5.5 to 7.5, further highlighting the importance of charge-based interactions with PA for the defensin to permeabilise fungal cells (Fig. 5d).

As shown in Fig. 2d, the binding of PA also triggers the formation of CpoBD13:PA oligomers. When observed using TEM (Fig. 2e), the CpoBD13:PA oligomeric complexes resembled clusters of long, sheet-like structures; a morphology vastly different to the defensin–lipid fibrils produced by the plant defensins NaD1 and NsD7^10,11^. As there are no other reported TEM images of β-defensin:lipid oligomers to date, it is currently unknown if the CpoBD13:PA sheet-like morphology observed is representative for β-defensins. However, it is likely that these structures are exaggerated versions of smaller physiological membrane disruption complexes that are generated only within localised regions of the fungal membrane upon accumulation of CpoBD13 to those regions enriched with PA. Examination of the crystal structure of the CpoBD13:PA complex and the presence of a continuous left-handed single-stranded helical chain formed between the defensin protomers raises the possibility that multiple single strands could orientate parallel to one another, forming the observed oligomeric sheets. It remains unclear whether the oligomerisation of CpoBD13 is a crucial step in the membrane permeabilisation of target cells as there was no clear difference in oligomerisation between the assessed mutants (Fig. 4h), even for CpoBD13 (H21A) that showed the greatest reduction in membranolytic activity.

Overall, our findings reveal that the structure and lipid-binding capability of β-defensins have been conserved from the divergence of humans and reptiles to the present day. Intriguingly, the structure of our CpoBD13:PA complex revealed the existence of large and continuous helical oligomers. Whilst phospholipid binding has been previously shown for other *trans*-defensins such as HBD-2 and HBD-3, the atomic architecture of postulated *trans*-defensin:phospholipid oligomeric membrane attack complexes remained unresolved to date. Our structural analysis of CpoBD13:PA provides a first glimpse into the architecture of a *trans*-defensin:phospholipid oligomeric complex. Considering the conserved location of key interacting residues between HBD-2 and CpoBD13, this may provide a clue as to the possible architecture of HBD-2 oligomeric assemblies or those involving other related *trans-*defensins. Our findings suggest that despite having arisen independently during evolution, both *cis*- and *trans*-defensins are capable of forming highly ordered large oligomeric membrane permeabilising structures with specific phospholipids. Furthermore, the observed pH-dependent membranolytic activity of CpoBD13 suggests that reptiles are capable of regulating defensin activities using a naturally occurring ‘histidine-switch’ mechanism, whereby the charge of histidine residues can be utilised to turn antifungal activity on and off depending on the environmental pH. These data may inform the engineering of pH-dependent CHDPs for therapeutic purposes.

## Methods

### Cloning and recombinant expression of CpoBD13, CpoBD13 mutants and CpoBD13-HA

The open reading frame encoding CpoBD13 was identified from the NCBI nucleotide database as accession XM_019537183.1. The DNA sequence encoding the mature defensin domain of CpoBD13 (nucleotides 142–255), as determined using the web-based software SignalP^24^, or with an additional C-terminal haemagglutinin (HA) tag (YPYDVPDYA) was codon-optimised for expression in the methylotrophic yeast *Pichia pastoris*, synthesised by Bioneer Pacific (Victoria, Australia), subcloned into the pPIC9 vector for recombinant protein expression in *P. pastoris*, and subsequently purified by cation exchange chromatography, as previously described^25^. CpoBD13 point mutations (R9A, R17A, H21A, H33A and H35A) were generated using the QuikChange II Site-Directed Mutagenesis Kit (Agilent, Santa Clara, CA) as per the manufacturer’s instructions. CpoBD13 compound mutants were synthesised by GenScript Biotech (Nanjing, China) directly into the pPIC9 vector.

### Fungal growth inhibition and propidium iodide uptake assays

For fungal growth assays, overnight cultures of *C. albicans* ATCC 10231 cells (yeast peptone dextrose broth, 30°C, shaking at 160 rpm) were pelleted (2000 *g*, 5 min), washed once in 0.5× potato dextrose broth (BD Biosciences, Franklin Lakes, NJ) and finally resuspended in the same media. Cells were seeded onto a 96-well plate (400 cells, 50 µL) and treated with an equal volume of varying concentrations of defensin in 0.5× potato dextrose broth. After incubating the samples (24 h, 30 °C), the plates were shaken to distribute the cells and growth was determined by performing a 9-well scan (600 nm) using a Spectramax M5e microplate reader (Molecular Devices, San Jose, CA). For assays testing defensin salt tolerance, *C. albicans* cells were instead resuspended in 1x potato dextrose broth and treated with an equal volume of CpoBD13 + NaCl solution at their intended concentrations. Propidium iodide (PI) uptake assays were performed by preparing the cells as per the standard growth inhibition assay however they were instead seeded onto a U-bottom 96-well plate (50,000 cells, 25 µL). After incubating the cells with an equal volume of defensin in 0.5× potato dextrose broth at a range of concentrations (30 min, shaking at 300 rpm), PI (diluted in ice-cold PBS) was added to the samples (final concentration of 1 µg/mL) before analysis by flow cytometry using a BD FACSCanto II with FACSDiva 8.0.1 (BD Biosciences). For PI uptake experiments performed at a specific pH, the same method was followed with the exception that cells were resuspended in 1× potato dextrose broth while defensin titrations were diluted in 40 mM MES buffer at the intended pH. An example PI uptake gating strategy is presented in Supplementary Fig. 1d.

### Cell lines and cultures

Human epithelial cervical cancer (HeLa, ATCC, CCL-2), prostate cancer (PC3, ATCC, CRL-1435) and histiocytic lymphoma (U937, ATCC, CRL-1593.2) cells were obtained from the American Type Culture Collection (Gaithersburg, Maryland) and cultured in RPMI-1640 medium (Thermo Fisher Scientific, Waltham, MA) supplemented with 5–10% (v/v) foetal bovine serum (FBS), 100 U/mL penicillin and 100 U/mL streptomycin (Invitrogen, Carlsbad, CA). Adult human dermal fibroblasts (AHDF, Lonza, CC-2511) cells were obtained from Lonza (Basel, Switzerland) and cultured in Dulbecco’s Modified Eagle Medium (DMEM) (Thermo Fisher Scientific) supplemented with 10% (v/v) FBS, 100 U/mL penicillin and 100 U/mL streptomycin. Human umbilical vein epithelial (HUVEC, Lonza, CC-2519) cells were obtained from Lonza and cultured in EBM-2 medium supplemented with EGM-2 SingleQuots (Lonza). All cell lines were maintained at 37 °C in a humidified incubator with 5% CO_2_.

### Mammalian cell viability and propidium iodide uptake assays

Cell viability assays were performed using MTT/MTS as previously described^8^. For propidium iodide uptake assays in serum-free medium, U937 cells (after centrifugation at 300 *g*, 5 min) were washed once in serum-free RPMI-1640, pelleted again (as previous centrifugation) and resuspended in serum-free media. 4×10^4^ cells were seeded and incubated with a range of CpoBD13 concentrations (diluted in serum-free medium) for 30 min at 37°C. PI (diluted in ice-cold PBS) was added to a final concentration of 1 µg/mL before analysis by flow cytometry using a BD FACSCanto II with FACSDiva 8.0.1 (BD Biosciences).

### BODIPY labelling of CpoBD13 carboxyl groups

Lyophilised CpoBD13 (2 mg) was resuspended in 900 µL of conjugation buffer (100 mM MES, 500 mM NaCl, pH 6.0) followed by the addition of 0.71 mg 1-ethyl-3-(3-dimethylaminopropyl)carbodiimide (EDC) and 2 mg N-hydroxysuccinimide (50 µL of each, dissolved in conjugation buffer). After incubating at RT for 15 min, the pH of the reaction was adjusted to between 7–8 by the addition of 20× PBS. BODIPY FL EDA (Thermo Fisher Scientific) was resuspended in dimethylsulfoxide to a concentration of 20 mg/mL before it was subsequently added to the reaction mixture (41 µL, molar excess of 5:1). After incubating the reaction for 150 min, salts and unbound BODIPY were removed by using a PD-10 desalting column (Cytiva, Marlborough, MA) as per the manufacturer’s instructions.

### Confocal laser scanning microscopy

Live cell imaging was performed on a Zeiss LSM 800 confocal microscope using a 63× oil immersion objective at 30 °C in 5% CO_2_. *C. albicans* ATCC 10231 cells suspended in 0.5× potato dextrose broth were immobilised onto the surface of glass chamber wells pre-coated with 0.01% poly-L-lysine (Sigma-Aldrich, Burlington, MA) and were treated with CpoBD13-BODIPY (20 µM) immediately prior to imaging. For microscopy experiments containing PI, the dye was added to the cell media to give a final concentration of 1 µg/mL.

### Protein–lipid overlay assay

PIP Strips (Echelon Biosciences, Salt Lake City, UT) were initially incubated in PBS/3% BSA for 60 min. The blocked strips were then incubated with CpoBD13-HA (1 µg/mL) diluted in PBS with 1% BSA for 60 min followed by three 10 min washes with PBS/0.1% Tween-20 at RT. Bound CpoBD13-HA was probed for using rabbit anti-HA tag antibody (ab9110, Abcam, Cambridge, United Kingdom) diluted to 1:4000 in PBS/1% BSA for 120 min at RT. The PIP Strips were washed before being incubated with HRP-conjugated donkey anti-rabbit Ig (NA934, GE Healthcare, Buckinghamshire, United Kingdom) diluted to 1:5000 in PBS/1% BSA for 60 min at RT. Following a final round of washing, antibody reactivity was detected by chemiluminescence using the ECL Prime Western Blotting System (Cytiva) as per the manufacturer’s instructions.

### Chemical crosslinking

CpoBD13 (1 mg/mL, 5 µL) was incubated with an equivalent volume of 0, 0.092 or 0.46 mM 08:00 phosphatidic acid (PA) (Avanti Polar Lipids, Alabaster, AL) in 20 mM HEPES pH 7.1 for 30 min before the addition of equal parts 20 mM PEGylated bis(sulfosuccinimidyl) suberate (BS(PEG)_5_, Thermo Fisher Scientific) in the aforementioned buffer solution. After a 30 min incubation, samples were subjected to SDS-PAGE analysis and colloidal Coomassie staining. Crosslinking experiments with CpoBD13 mutants were performed using the same method however only a single PA concentration (0.46 mM) was assessed.

### Liposome pulldown/lysis assays

Liposomes were prepared using natural chicken egg L-α-phophatidylcholine and L-α-phosphatidic acid dissolved in chloroform (Avanti Polar Lipids), and liposome pulldowns (at pH 7.4) and calcein-encapsulated liposome lysis assays were performed as previously described^6^. For liposome pulldowns performed at pH 5.5, the same protocol was used with minor variations. The desiccated lipid films were resuspended in 20 mM MES pH 7.4 and were washed in the same buffer during the centrifugation process. After the final wash step, the liposome pellets were resuspended in 20 mM MES pH 5.5 and incubated with CpoBD13 or mutants thereof (also prepared in the same acidic buffer).

### Transmission electron microscopy (TEM)

Samples were prepared by mixing 1 mg/mL CpoBD13 with 4 mM PA or PC at a 9:1 ratio (final molar ratio of 1:1). For samples of CpoBD13, PA or PC only, protein/lipid was diluted in water to give the same concentration as the CpoBD13 + lipid sample. Samples (10 µL) were allowed to settle onto 400-mesh copper grids before excess liquid was removed with blotting paper. Each sample was negative stained twice with 2% uranyl acetate, allowed to air-dry and imaged using a JEOL JEM-2010 transmission electron microscope (JEOL, Akishima, Japan). For experiments in reducing conditions, CpoBD13 and PA were mixed in the presence of 100 mM dithiothreitol (DTT) prior to negative staining.

### X-ray crystallography

Crystals of CpoBD13 were grown at 20°C in 0.1 M trisodium citrate pH 5.5, 20% PEG 3000 using protein at a concentration of 20 mg/mL. Crystals of CpoBD13:PA were grown at 20°C in 0.8 M Na_2_HPO_4_/KH_2_PO_4_, 0.1 M sodium HEPES pH 7.5 using a protein:lipid molar ratio of 1:1.2 at a concentration of 14 mg/mL. Diffraction data were collected on the MX2 beamline at the Australian Synchrotron. All diffraction data were integrated using Dials^26^ or Xia2^27^ version 3.2.1. and scaled using AIMLESS version 0.7.4^28,29^. The structure of CpoBD13 was solved by molecular replacement with PHASER^30^ using the structure of HBD-2 (PDB ID 6CS9)^6^ as a search model. The structure of CpoBD13:PA was solved by molecular replacement with PHASER^30^ using the previously determined structure of CpoBD13. Final models were manually built using *Coot*^31^ and refined using *PHENIX* version 1.18.2_3874^32^. All data collection and refinement statistics are summarised in Supplementary Table 1. Representative images of the electron density maps for both structures are presented in Supplementary Fig. 3.

### Statistical analysis

All data are presented as the mean ± SEM of three independent experiments. Where stated, statistical analyses of these experiments (including two-way ANOVA and unpaired t-tests) were performed using Prism 9.1 (GraphPad, San Diego, CA).

### Reporting summary

Further information on research design is available in the Nature Portfolio Reporting Summary linked to this article.

## Supplementary information

Supplementary Information

Description of Additional Supplementary Files

Supplementary Movie 1

Reporting Summary

## Data Availability

The data that support this study are available from the corresponding authors upon reasonable request. Coordinate files have been deposited in the Protein Data Bank (PDB) under the accession codes 7T9R and 7T9Q. Previously published structures from the PDB can be accessed with accession code 1FD3 (HBD-2). Sequence alignment of CpoBD13 with its close homologues *A. sinensis* (Chinese alligator) β-defensin 13 (AsBD13) [https://www.ncbi.nlm.nih.gov/protein/AUG31287.1] and *G. gallus* (chicken) avian β-defensin 13 (AvBD13) [https://www.ncbi.nlm.nih.gov/protein/NP_001001780.1]. All raw diffraction images were deposited in the SBGrid Data Bank^33^. The source data underlying Figs. 1c–g, 2a–d, 4a–h, 5b–d and Supplementary Figures 1a, b are provided as a Source Data file. Source data are provided with this paper.

## Source data

Source Data
