# Supplementary material for: Crocodile defensin (CpoBD13) antifungal activity via pH-dependent phospholipid targeting and membrane disruption
Source: Nat Commun. 2023 Mar 1;14:1170. doi: 10.1038/s41467-023-36280-y (PMC9977887; doi:10.1038/s41467-023-36280-y)
Supplement: Supplementary file 1 — Supplementary Information [file 41467_2023_36280_MOESM1_ESM.pdf]

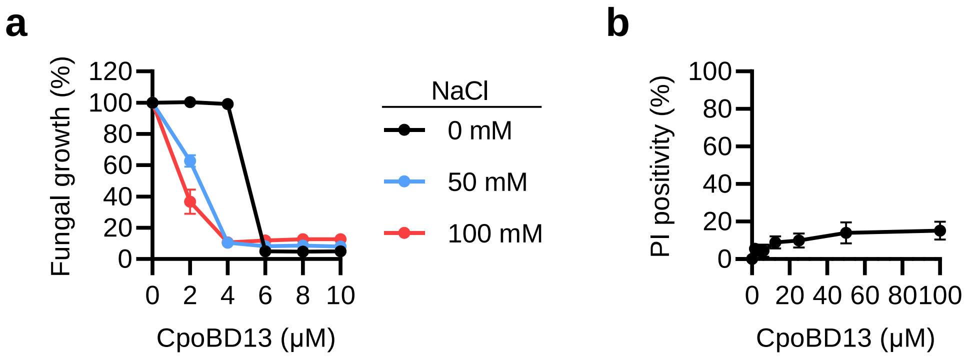

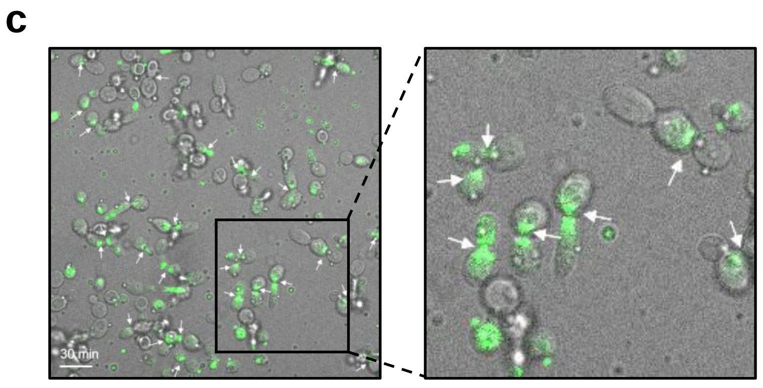

Total cells	123
Total cell-cell contacts	89
BODIPY positive cell-cell contacts	28
% BODIPY positive cell-cell contacts	31.5

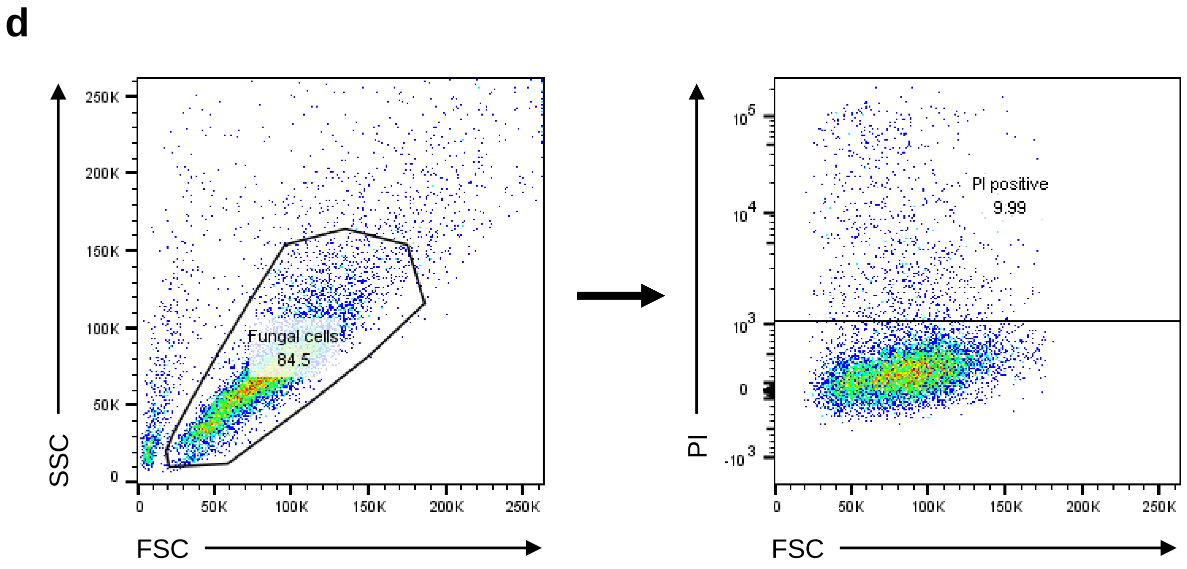

Supplementary Fig. 1. a Fungal growth inhibition of *C. albicans* treated with a range of CpoBD13 in various concentrations of NaCl over 24 h. Data represents mean ± SEM, n=3. **b** Membrane permeabilisation of mammalian U937 cells treated with a range of CpoBD13 concentrations in serum-free medium over 30 min as determined by the uptake of propidium iodide. Data represents mean ± SEM, n=3. **c** Quantification of CpoBD13-BODIPY accumulation at *C. albicans* cell to cell contact points as observed in Fig. 1i and Supplementary Movie 1. **d** Example propidium iodide uptake assay gating strategy. **a** & **b** Source data are provided as a Source Data file.

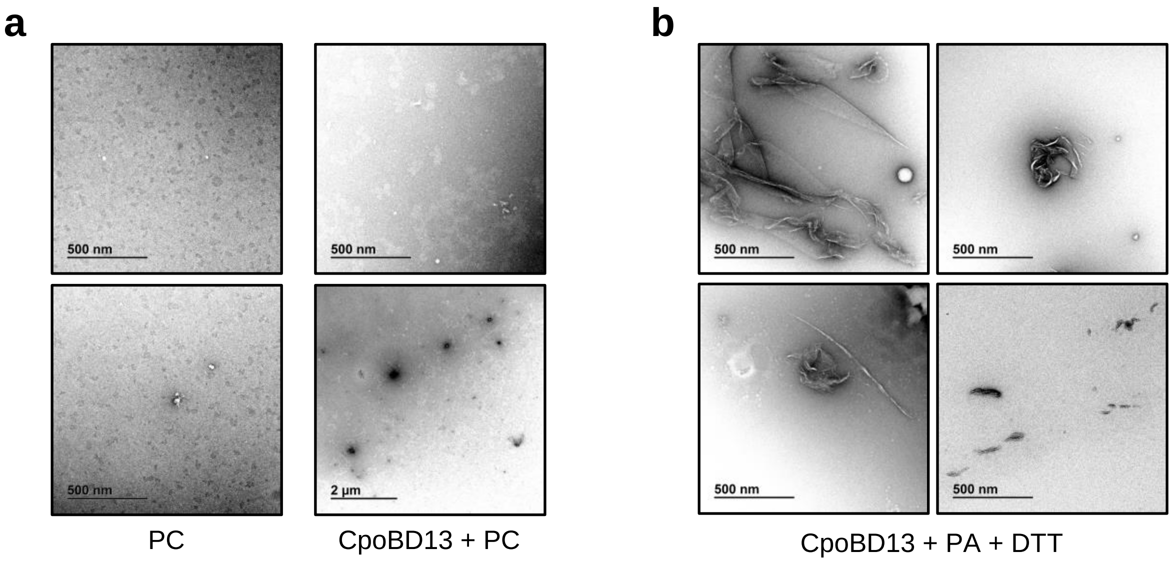

Supplementary Fig. 2. a TEM images of PC alone and CpoBD13 incubated with PC. **b** TEM images of CpoBD13-PA complexes produced in the presence of the reducing agent dithiothreitol (DTT). Images in **a** & **b** are representative fields of one independent experiment.

a

b

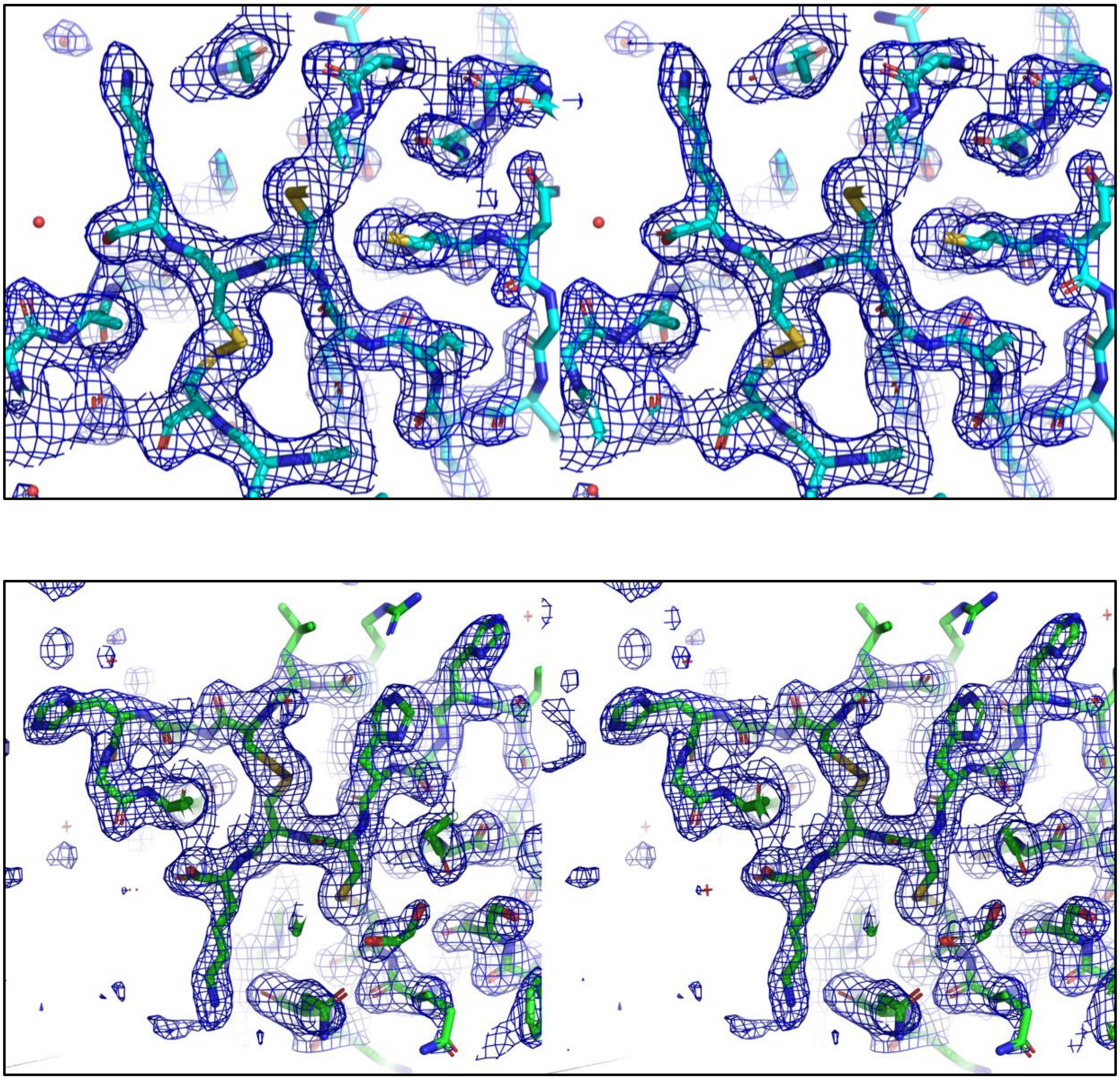

Supplementary Fig. 3. Electron density map of a representative section of **a** CpoBD13 (shown as cyan sticks) and **b** the CpoBD13:PA complex (shown as green sticks). Maps are a 2Fo-Fc map contoured at 1 σ shown as blue mesh. Wall-eye stereo representation.

Supplementary Table 1.
Crystallographic data collection and refinement statistics of CpoBD13 and CpoBD13:PA

	CpoBD13	CpoBD13:PA
**Data collection**		
Space group	$C222_1$	P1
Cell dimensions		
a, *b*, *c* (Å)	46.70, 56.42, 33.10	27.06, 33.69, 36.64
α, β, γ (°)	90.00, 90.00, 90.00	90.09, 90.02, 90.01
Wavelength (Å)	0.9537	0.9537
Resolution (Å)*	19.08-1.8 (1.865-1.80)	24.82-1.45 (1.48-1.45)
R_{sym} or R_{merge}*	0.085 (0.521)	0.096 (0.922)
$I / \sigma I$*	4.2 (1.1)	6.5 (2.0)
Completeness (%)*	98.0 (99.3)	93.0 (90.3)
Redundancy*	2.7 (2.8)	2.5 (2.5)
CC(1/2)	0.995 (0.461)	0.990 (0.416)
**Refinement**		
Resolution (Å)	19.08-1.8	24.78-1.45
No. reflections	4149	21266
R_{work} / R_{free}	0.227/0.253	0.194/0.215
No. atoms		
Protein	316	1274
Ligand/ion	19	101
Water	22	130
B-factors		
Protein	37.7	15.0
Ligand/ion	58.0	35.0
Water	48.6	23.9
R.m.s. deviations		
Bond lengths (Å)	1.18	1.06
Bond angles (°)	0.011	0.006
Molprobity score	1.46	1.55

*Values in parentheses are for highest-resolution shell.
