# Supplementary material for: Crocodile defensin (CpoBD13) antifungal activity via pH-dependent phospholipid targeting and membrane disruption
Source: Nat Commun. 2023 Mar 1;14:1170. doi: 10.1038/s41467-023-36280-y (PMC9977887; doi:10.1038/s41467-023-36280-y)
Supplement: Supplementary file 2 — Description of Additional Supplementary Files [file 41467_2023_36280_MOESM2_ESM.pdf]

Description of Additional Supplementary Files

File Name: Supplementary Movie 1
Description: Live microscopy of the accumulation of CpoBD13-BODIPY (20 µM, green) at the plasma membrane of C. albicans cells. Arrows indicate cell to cell contact points where CpoBD13-BODIPY accumulation is present. Scale bar represents 10 µm.
