# Supplementary material for: Crocodile defensin (CpoBD13) antifungal activity via pH-dependent phospholipid targeting and membrane disruption
Source: Nat Commun. 2023 Mar 1;14:1170. doi: 10.1038/s41467-023-36280-y (PMC9977887; doi:10.1038/s41467-023-36280-y)
Supplement: Supplementary file 4 — Reporting Summary [file 41467_2023_36280_MOESM4_ESM.pdf]

nature portfolio

Corresponding author(s): DBPR NCOMMS-22-11166B

Last updated by author(s): 2023-1-16

Reporting Summary

Nature Portfolio wishes to improve the reproducibility of the work that we publish. This form provides structure for consistency and transparency in reporting. For further information on Nature Portfolio policies, see our Editorial Policies and the Editorial Policy Checklist.

Statistics

For all statistical analyses, confirm that the following items are present in the figure legend, table legend, main text, or Methods section.

n/a	Confirmed	
☐	☒	The exact sample size (*n*) for each experimental group/condition, given as a discrete number and unit of measurement
☐	☒	A statement on whether measurements were taken from distinct samples or whether the same sample was measured repeatedly
☐	☒	The statistical test(s) used AND whether they are one- or two-sided<br>*Only common tests should be described solely by name; describe more complex techniques in the Methods section.*
☒	☐	A description of all covariates tested
☒	☐	A description of any assumptions or corrections, such as tests of normality and adjustment for multiple comparisons
☐	☒	A full description of the statistical parameters including central tendency (e.g. means) or other basic estimates (e.g. regression coefficient) AND variation (e.g. standard deviation) or associated estimates of uncertainty (e.g. confidence intervals)
☐	☒	For null hypothesis testing, the test statistic (e.g. *F*, *t*, *r*) with confidence intervals, effect sizes, degrees of freedom and *P* value noted<br>*Give P values as exact values whenever suitable.*
☒	☐	For Bayesian analysis, information on the choice of priors and Markov chain Monte Carlo settings
☒	☐	For hierarchical and complex designs, identification of the appropriate level for tests and full reporting of outcomes
☒	☐	Estimates of effect sizes (e.g. Cohen's *d*, Pearson's *r*), indicating how they were calculated

Our web collection on statistics for biologists contains articles on many of the points above.

Software and code

Policy information about availability of computer code

Data collection	Diffraction data were collected on the MX2 beamline at the Australian Synchrotron.<br>Live cell imaging was performed on a Zeiss LSM 800 confocal microscope using a 63x oil immersion objective.<br>Flow cytometry was performed using a BD FACSCanto II (BD Biosciences).
Data analysis	All diffraction data were integrated using Xia2 version 3.2.1. and merged using AIMLESS version 0.7.4. The structures of CpoBD13 and CpoBD13:PA were solved by molecular replacement with PHASER, built using Coot and refined using PHENIX as implemented in Phenix Version 1.18.2_3874.<br>Immunofluorescence images were analyzed using Zeiss ZEN version 3.6.<br>Flow cytometry data was analyzed using BD FACSDiva 8.0.1 and FloJo 10.7.1.

For manuscripts utilizing custom algorithms or software that are central to the research but not yet described in published literature, software must be made available to editors and reviewers. We strongly encourage code deposition in a community repository (e.g. GitHub). See the Nature Portfolio guidelines for submitting code & software for further information.

Data

Policy information about availability of data

All manuscripts must include a data availability statement. This statement should provide the following information, where applicable:

- Accession codes, unique identifiers, or web links for publicly available datasets
- A description of any restrictions on data availability
- For clinical datasets or third party data, please ensure that the statement adheres to our policy

Crystallographic data have been deposited with the Protein Data Bank using accession codes 7T9R and 7T9Q. This includes the coordinate files as well as the diffraction data file against which the refinements were performed. https://www.rcsb.org/structure/unreleased/7T9Q, https://www.rcsb.org/structure/unreleased/7T9R.
Data availability statement as provided in the manuscript: Coordinate files have been deposited in the Protein Data Bank under the accession codes 7T9R [https://www.rcsb.org/structure/unreleased/7T9R] and 7T9Q [https://www.rcsb.org/structure/unreleased/7T9Q].
All raw diffraction images were deposited in the SBGrid Data Bank. The source data underlying Figures 1C-G, 2A-D, 4A-H, 5B-D and Supplementary Figures 1A-B are provided as a Source Data file.

Human research participants

Policy information about studies involving human research participants and Sex and Gender in Research.

Reporting on sex and gender	N/A
Population characteristics	N/A
Recruitment	N/A
Ethics oversight	N/A

Note that full information on the approval of the study protocol must also be provided in the manuscript.

Field-specific reporting

Please select the one below that is the best fit for your research. If you are not sure, read the appropriate sections before making your selection.

☒ Life sciences ☐ Behavioural & social sciences ☐ Ecological, evolutionary & environmental sciences

For a reference copy of the document with all sections, see nature.com/documents/nr-reporting-summary-flat.pdf

Life sciences study design

All studies must disclose on these points even when the disclosure is negative.

Sample size	No statistical methods were used to predetermine sample size. All experiments for calculation of statistical significance were performed using at least three or more biological replicates consistent with our similar previously published studies and that of others. The only exception was for transmission electron microscopy where two biological replicates were performed and multiple sample regions and individual fields analyzed (at least >10 of each).
Data exclusions	No data was excluded from the analyses.
Replication	All attempts at replication were successful.
Randomization	Randomization was not performed for this study. Cell or protein samples were allocated to experimental groups and analyzed in comparison to controls under identical conditions.
Blinding	The investigators were not blinded in this study during group allocation and data analysis. To eliminate bias and ensure prior knowledge had no influence on generated data, all samples and control groups for experiments were prepared and analyzed at the same time under identical conditions.

Reporting for specific materials, systems and methods

We require information from authors about some types of materials, experimental systems and methods used in many studies. Here, indicate whether each material, system or method listed is relevant to your study. If you are not sure if a list item applies to your research, read the appropriate section before selecting a response.

Materials & experimental systems

n/a	Involved in the study
☐	☒ Antibodies
☐	☒ Eukaryotic cell lines
☒	☐ Palaeontology and archaeology
☒	☐ Animals and other organisms
☒	☐ Clinical data
☒	☐ Dual use research of concern

Methods

n/a	Involved in the study
☒	☐ ChIP-seq
☐	☒ Flow cytometry
☒	☐ MRI-based neuroimaging

Antibodies

Antibodies used	Rabbit anti-HA (Abcam, ab9110), HRP-conjugated donkey anti-rabbit (Sigma-Aldrich, NA934).
Validation	Antibodies were validated by the manufacturers and cited extensively in the literature as detailed: [https://www.abcam.com/products?keywords=rabbit%20anti%20HA%209110]; [https://www.sigmaaldrich.com/AU/en/product/sigma/gena9341ml].

Eukaryotic cell lines

Policy information about cell lines and Sex and Gender in Research

Cell line source(s)	Human epithelial cervical cancer (HeLa) #CCL-2, prostate cancer (PC3) #CRL-1435 and histiocytic lymphoma (U937) #CRL-1593.2 were acquired from ATCC. Human umbilical vein epithelial (HUVEC) #CC-2519 and Adult human dermal fibroblast (AHDF) #CC-2511 and were acquired from Lonza.
Authentication	HeLa, PC3 and U937 were authenticated using short tandem repeat (STR) profiling. HUVEC were authenticated by cell surface marker analysis using flow cytometry [https://www.nature.com/articles/s41598-017-14305-z]. AHDF were not authenticated.
Mycoplasma contamination	All cell lines were tested for mycoplasma contamination by PCR and were negative.
Commonly misidentified lines (See ICLAC register)	No commonly misidentified cell lines were used in this study.

Flow Cytometry

Plots

Confirm that:

☒ The axis labels state the marker and fluorochrome used (e.g. CD4-FITC).

☒ The axis scales are clearly visible. Include numbers along axes only for bottom left plot of group (a 'group' is an analysis of identical markers).

☒ All plots are contour plots with outliers or pseudocolor plots.

☒ A numerical value for number of cells or percentage (with statistics) is provided.

Methodology

Sample preparation	U937 cells were collected by centrifugation at 300 x g. Harvested cells were washed by resuspension in serum-free RPMI-1640 and centrifugation at 300 x g. U937 cell pellets were resuspended for flow cytometry analysis in serum-free RPMI-1640. C. albicans was pelleted by centrifugation at 2000 x g, washed once in 0.5x potato dextrose broth and resuspended in the same media for analysis.
Instrument	Analysis by flow cytometry was performed using a BD FACSCanto II (BD Biosciences).
Software	The software use to collect and analyze the the flow cytometry data was BD FACSDiva 8.0.1 and FloJo 10.7.1.
Cell population abundance	Ten thousand cells were analysed and PI positive cells ranged from 0% to > 90% depending on the amount and time duration of defensin treatment.
Gating strategy	PI positive and negative cell populations were gated as illustrated in Supplementary Fig. 1D.

☒ Tick this box to confirm that a figure exemplifying the gating strategy is provided in the Supplementary Information.
